# State of the Art in the Development of Human Serum Carnosinase Inhibitors

**DOI:** 10.3390/molecules29112488

**Published:** 2024-05-24

**Authors:** Luca Regazzoni

**Affiliations:** Department of Pharmaceutical Sciences, University of Milan, Via Mangiagalli 25, 20133 Milan, Italy; luca.regazzoni@unimi.it; Tel.: +39-02-503-19340

**Keywords:** serum carnosinase, carnosine, inhibitors, carnostatine, bestatin

## Abstract

Human serum carnosinase is an enzyme that operates the preferential hydrolysis of dipeptides with a C-terminus histidine. Only higher primates excrete such an enzyme in serum and cerebrospinal fluid. In humans, the serum hydrolytic rate has high interindividual variability owing to gene polymorphism, although age, gender, diet, and also diseases and surgical interventions can modify serum activity. Human genetic diseases with altered carnosinase activity have been identified and associated with neurological disorders and age-related cognitive decline. On the contrary, low peripheral carnosinase activity has been associated with kidney protection, especially in diabetic nephropathy. Therefore, serum carnosinase is a druggable target for the development of selective inhibitors. However, only one molecule (i.e., carnostatine) has been discovered with the purpose of developing serum carnosinase inhibitors. Bestatin is the only inhibitor reported other than carnostatine, although its activity is not selective towards serum carnosinase. Herein, we present a review of the most critical findings on human serum carnosinase, including enzyme expression, localization and substrate selectivity, along with factors affecting the hydrolytic activity, its implication in human diseases and the properties of known inhibitors of the enzyme.

## 1. Enzymatic Activity of Human Serum Carnosinase

### 1.1. Enzyme Classification

Human serum carnosinase is an alternative name for the enzyme beta alanyl-histidine dipeptidase. Other synonyms found in literature or in some enzyme databases (e.g., ExplorEnz, BRENDA, IUBMB Enzyme Nomenclature, PRIAM enzyme-specific profiles) are CN1, CNDP1, carnosinase, serum carnosinase, carnosinase-1, and carnosine dipeptidase 1.

**Figure 1:**
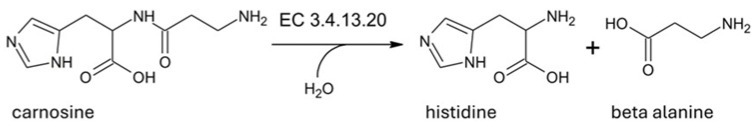
Hydrolysis reaction catalyzed by human serum carnosinase (EC 3.4.13.20).

The name beta alanyl-histidine dipeptidase has been assigned according to EC nomenclature, which classifies enzymes according to the reaction catalyzed, and is recommended by the International Union of Biochemistry and Molecular Biology (IUBMB, https://iubmb.qmul.ac.uk/enzyme/, accessed on 1 April 2024) [1]. The code assigned to human serum carnosinase is EC 3.4.13.20, which classifies the enzyme as a member of the hydrolase family (i.e., EC 3), acting on peptide bonds (i.e., peptidases, EC 3.4), with a selectivity on dipeptides (i.e., dipeptidases, EC 3.4.13). Among all dipeptidases, the full code EC 3.4.13.20 specifically identifies the enzyme operating preferential hydrolysis of the dipeptide carnosine, according to the reaction reported in Figure 1.

For this reason, some synonyms of the enzyme include the term carnosinase (e.g., serum carnosinase, carnosinase-1). However, since carnosine is an alternative name for the dipeptide beta alanyl-histidine, the recommended enzyme name is beta alanyl-histidine dipeptidase.

### 1.2. Closely Related Enzymes

Since another human dipeptidase catalyzes carnosine hydrolysis, some of the synonyms of serum carnosinase include the number one (e.g., carnosinase-1, carnosine dipeptidase 1, CN1). The existence of two forms of human carnosinase was first detected in human tissue extracts, upon electrophoretic separation of two distinct peaks with carnosinase activity [2]. However, it took three decades to characterize the genes, the tissue localization, and the features of the two enzymes [3,4]. Since their identification and distinction, the two enzymes have been reported as carnosinase-1 (also known as CNDP1, CN1, carnosine dipeptidase 1, serum carnosinase, EC 3.4.13.20) and carnosinase-2 (also known as CNDP2, CN2, carnosine dipeptidase 2, cytosolic non-specific dipeptidase, EC 3.4.13.18).

Alternative identification codes for carnosinases are reported by the MEROPS database, which was created to classify enzymes according to their structure. Carnosinases are included in the MH clan and M20 family with the codes M20.006 (carnosine dipeptidase 1) and M20.005 (carnosine dipeptidase 2). Such a family contains exopeptidases that bind two metal ions per monomer of protein (e.g., carboxypeptidases, dipeptidases and the specialized bacterial aminopeptidase called peptidase T) [5].

**Figure 2:**
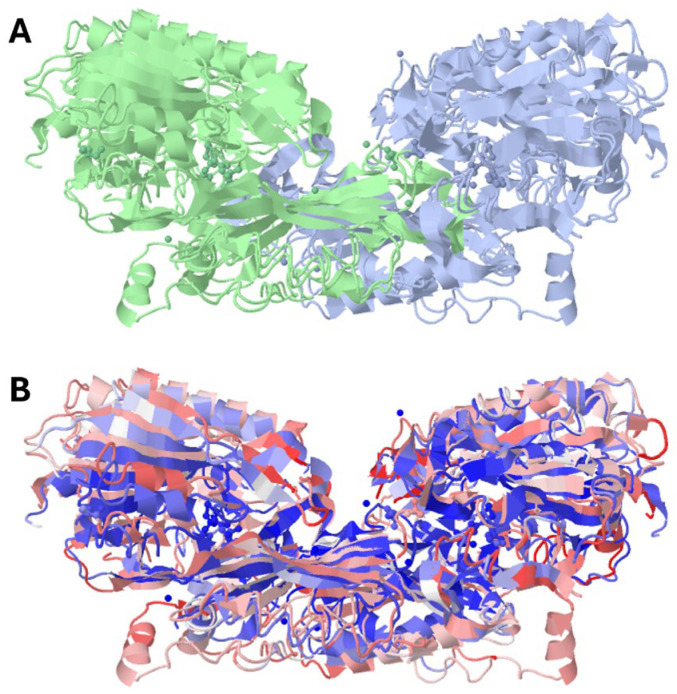
Crystal structure of human carnosine dipeptidase 1 (PDB id: 3dlj) overlapped with the crystal structure of human carnosine dipeptidase 2 (PDB id: 4ruh). (**A**) reports the two monomers with separate colors; (**B**) reports carnosine dipeptidase 1 in red and carnosine dipeptidase 2 in blue.

As recently reported, human carnosinases share 49% of their primary sequence and have a similar homodimeric structure to that depicted in Figure 2.

**Figure 3:**
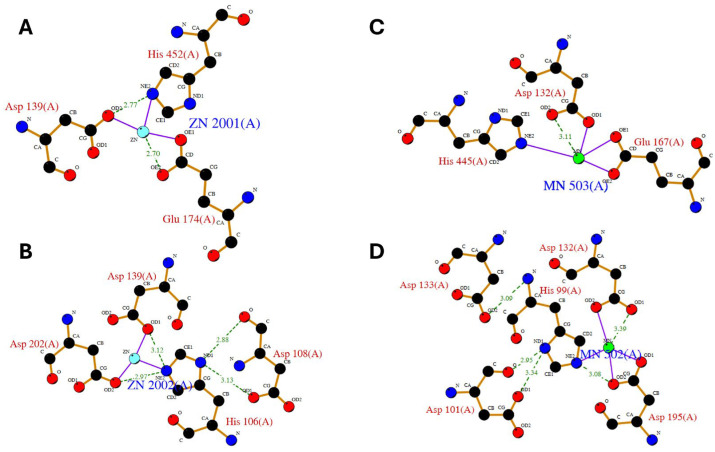
LIGPLOT diagram of main zinc interactions with human carnosine dipeptidase 1 (**A**,**B**) as from enzyme crystal structure (PDB id: 3dlj) and LIGPLOT diagram of main manganese interactions with human carnosine dipeptidase 2 (**C**,**D**) as from enzyme crystal structure (PDB id: 4ruh).

However, the catalytic pockets of carnosinase-2 bind bivalent manganese, whereas carnosinase-1 binds zinc [3,6,7,8,9,10,11,12]. Despite such a difference, the crystal structure of the two enzymes reveals similar interactions with the metal ions, as reported by the LIGPLOT diagrams shown in Figure 3.

In addition to the type of metal ion required for activation, the two main functional features that separate human carnosinases are specified into the recommended EC name of carnosinase-2 (i.e., cytosolic non-specific dipeptidase). Such a name specifies that carnosinase-2 is located intracellularly (i.e., cytosolic dipeptidase), unlike carnosinase-1 that is mostly excreted in cerebrospinal fluid and serum [3,13,14]. A comparative experiment reporting the different localization of carnosinase-1 and carnosinase-2 was performed in mammal ovary cells transfected with either CN1 or CN2 human genes. Higher carnosine hydrolyzing activity was found in cell supernatant for CN1-transfected cells, while CN2-transfected cells have their maximum hydrolytic activity in the cytosol [3]. This confirms that human carnosinase-2 remains in the cytosol upon expression, whereas human carnosinase-1 is excreted. Interestingly, carnosinase-2 mRNA is ubiquitously detectable in all human tissues, whereas carnosinase-1 mRNA is mainly expressed in brain cells [3]. For this reason, serum carnosinase is likely to be produced mainly in the human brain and then excreted into the cerebrospinal fluid and serum.

The recommended name for human carnosinase-2 also suggests that such an enzyme is not specific towards carnosine (i.e., non-specific dipeptidase). In fact, in the early stages of its characterization, the enzyme was classified by many authors as prolinase, an enzyme operating preferentially but not selectively on hydrophobic dipeptides [15,16]. Notably, some authors suggested that carnosine is not even a physiological substrate of human carnosinase-2 [3]. This conclusion is supported by the evidence that at physiological pH (i.e., 7.5–8.5), carnosinase-1 operates the hydrolysis of carnosine and homocarnosine [3,14], which are the two most abundant substrates found in human skeletal muscles and the brain, respectively [17]. 

On the contrary, no activity of human carnosinase-2 has been reported towards homocarnosine [3], while carnosine can be hydrolyzed only at non-physiological pH (i.e., 9.5) [3,18]. Moreover, the hydrolytic activity of carnosine-1 is higher than carnosinase-2, even when both enzymes are tested at their optimum pH [19]. These findings raise concerns about the in vivo role of human carnosinase-2 as an enzyme operating the hydrolysis of histidine dipeptides (e.g., carnosine, homocarnosine).

### 1.3. Expression and Localization

In humans, serum carnosinase activity is typically found in the serum and cerebrospinal fluid, with higher carnosinase activity in serum [13]. Experimental evidence of enzyme expression and carnosinase activity has also been collected on the human kidney [20], which is the main place where carnosinase-1 activity is detectable in rodents [3,21,22,23]. Interestingly, no trace of the orthologue of human serum carnosinase is found in the serum and brains of rodents. The main carnosinase activity in the brain of rodents seems to depend on the activity of cytosolic non-specific dipeptidase (i.e., carnosinase-2), which, unlike the human orthologue, can operate at physiological pH and is able to bind both zinc and manganese [3,18,21]. This finding demonstrates that rodents have a different histidine dipeptide metabolism, especially in the central nervous system.

The main structural feature that distinguishes human serum carnosinase from animal orthologues is the N-terminal signal peptide that can be found only in the primary sequence of the enzyme expressed by higher primates or by the Syrian golden hamster [14]. The signal peptide is paramount for protein excretion. This hypothesis is reinforced by the evidence that altered carnosinase activity in human serum is associated with mutations in the N-terminal signal peptide due to polymorphisms of the CNDP1 gene [24,25,26,27,28,29,30]. Some of the mutations are associated with differential glycation patterns that reduce secretion and alter serum hydrolytic activity [31]. Therefore, the lack of a signal peptide in the primary sequence of rodent enzymes is likely the reason why no carnosinase activity is detectable in the serum of such species. Notably, the prevalence of some mutations of the human signal peptide has been observed in specific populations [28,29,32,33] or associated with sex [30,34,35] and athletic abilities [36,37,38].

### 1.4. Substrate Selectivity

**Figure 4:**
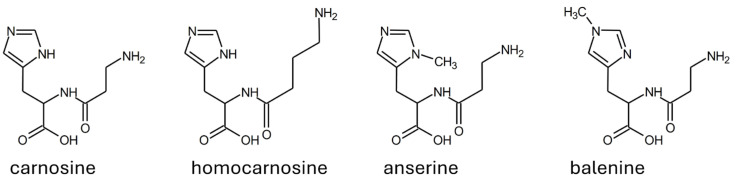
Structure of the most abundant substrates of human serum carnosinase.

As reported in Section 1.2, one distinctive trait of carnosinases is that carnosinase-2 is not selective. However, carnosinase-1 is also not uniquely specific for carnosine. In fact, the enzyme catalyzes the hydrolysis of other naturally occurring dipeptides with a C-terminus histidine or methylated histidine (i.e., homocarnosine, anserine, and balenine; see Figure 4) [3,4,14,39,40].

Moreover, other dipeptides with C-terminus leucine, phenylalanine, and alanine have been reported as substrates of serum carnosinase [14].

Although serum carnosinase does not operate the specific hydrolysis of one substrate only, the enzyme can be considered highly selective for carnosine on a kinetic base. In fact, the hydrolysis rate of dipeptides with a C-terminus histidine is faster than any other substrate, but carnosine kinetics is faster than any other dipeptide with a C-terminus histidine [3,14].

Concerning the naturally occurring dipeptides, the hydrolysis rate of homocarnosine and balenine is below 5% of the carnosine hydrolysis rate, while anserine has kinetics three-fold slower than carnosine [39,40,41]. Similarly, the non-natural dipeptide diaminopropionyl-L-histidine is hydrolyzed at 30% of the hydrolysis rate of carnosine [42].

Despite the fact that the faster hydrolysis of carnosine remains a consistent finding across the literature, some conflicting data have been reported for the absolute and relative hydrolysis rate of some substrates. Few examples concern how fast CN1 operates the hydrolysis of anserine, or if the enzyme can hydrolyze tripeptides containing histidine as the central amino acid [3,4,14,39]. The origin of such discrepancies can depend on the analytical methods used for the determination of hydrolytic rates. It has been recently suggested that fluorimetric methods based on OPA derivatization are not reliable when applied to methylated substrates such as anserine [39].

### 1.5. Substrate Recognition and Structure Activity Relationships

Over the years, many compounds have been tested as potential substrates of serum carnosinase. Such experimental activity was paralleled by several computational studies describing, in silico, the binding of serum carnosinase substrates [6,7,8,9,10,11,12]. As recently reviewed, the most recent computational models identified the main substrate features conducive for proper binding into the catalytic pocket as follows: (1) a carboxylate moiety interacting with Arg350, (2) a protonated amino group interacting with Asp139 and Asp202, (3) a peptide bond interacting with a zinc ion and Ser423, and (4) a group able to establish a H-bond with the neutral Glu173 located into a non-polar sub-pocket [12]. Such models explain the experimental evidence that carnosine has a faster hydrolysis rate compared with dipeptides with a methylated histidine (i.e., anserine, balenine), a different spatial pose of the N-terminus amino group (e.g., homocarnosine), or the C-terminus histidine replaced with leucine, phenylalanine, or alanine [14,39,40,42].

The in silico models were developed to be consistent with the experimental evidence that any alkylation of the N-terminus amine of carnosine (e.g., N-acetylcarnosine), as well as any modification or elimination of its C-terminus carboxylic moiety (e.g., carnosinol, carcinine), faces resistance to serum carnosinase [40,42,43,44,45]. Importantly, carcinine is not able to compete with carnosine or substrate binding [46]. Since the only structural difference between carainine and carnosine is the C-terminus carboxylic group, this finding is proof that such a group is essential for substrate recognition. However, no similar competition studies have been reported for the other derivatives of carnosine resistant to hydrolysis. Another important finding is that dipeptides with a non-natural orientation of the histidine chiral center (i.e., L configuration) are resistant to serum carnosinase. Experimental evidence was collected by testing the resistance of carnosine enantiomer (i.e., beta alanyl-D-histidine, also known as D-carnosine) [43,47]. Such a peptide can resist carnosinase hydrolysis because it has no access to the binding pocket or because no hydrolysis occurs upon its binding. Unfortunately, no experimental data support one of these two hypotheses.

The role of the two zinc ions was established based on similarity with other enzymes in the M20 family. The mechanistic hypothesis is that such ions are essential for the catalytic activity since they are involved in the stabilization of the tetrahedral intermediate between the substrate and the water molecule necessary for hydrolysis [3,6,7,8,9,10,11,12,48].

**Figure 5:**
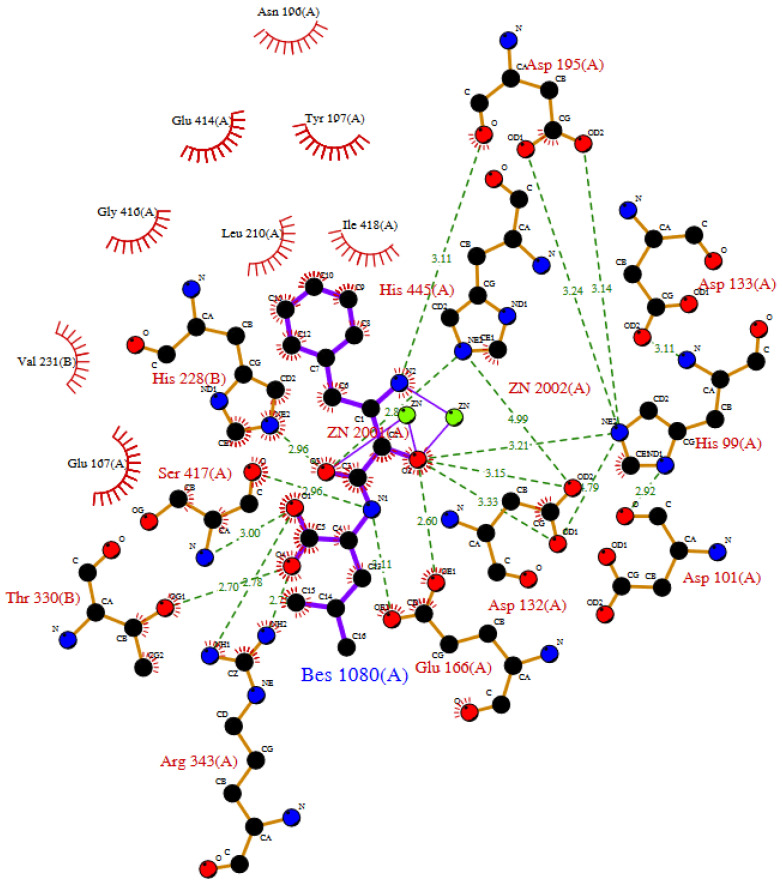
LIGPLOT diagram of mouse carnosinase 2 complexed with zinc and bestatin as from enzyme crystal structure (PDB id: 2zog).

Although no crystal structure of human serum carnosinase complexed with a substrate is available, the crystal structure of the complex between mouse carnosinase 2 complexed with zinc and a ligand (i.e., bestatin) is available (PDB id: 2zog). The main interactions of bestatin with mouse carnosinase 2 complexed with zinc are reposted in Figure 5. Such interactions are very similar to the interactions predicted by the computational models describing the interactions of carnosine with human serum carnosinase. Interestingly, mouse carnosinase 2 can operate carnosine hydrolysis (unlike its human orthologue, see Section 1.3), while bestatin is not hydrolyzed by CN1 or CN2 and inhibits both enzymes thanks to a structure that resembles the hydrolysis intermediate of carnosine (see in Section 2.1). 

In addition to a catalytic domain, serum carnosinase also has a dimerization domain, playing a key role for enzyme activity and substrate recognition [6,7,8,9,10,11,12]. The dimerization domain is important since the most abundant and active form of the enzyme in vivo is a homodimer, although a circulating monomeric enzyme has been detected as well. The monomeric form is prevalent in children, which can justify the observation of an increase with age in carnosinase activity, both in serum and cerebrospinal fluid [4,13,49,50]. Computer simulations suggest that the higher activity of the dimeric enzyme can depend on the interaction of the substrate with aminoacidic residues from both monomers [6,7,11,12]. 

Dimerization is, therefore, crucial for enzyme activity, as already reported for other enzymes in the M20 family [51]. Interestingly, recent findings suggest that the two catalytic pockets of serum carnosinase work independently and alternatingly [7,10]. Such a hypothesis is supported by kinetics data, showing that the occupation of both pockets is necessary for competitive inhibition of the enzyme [52].

Disulfide bond formation can contribute to homodimer stabilization. This was speculated since a change in the apparent molecular weight was detected by SDS-PAGE upon treatment with disulfide reducing agents [14]. However, such results are inconsistent since dimeric forms of serum carnosinase resistant to reducing agents have been detected as well [13].

Glycosylation is another structural feature with a significant impact on enzymatic activity. Serum carnosinase has been characterized as a glycoprotein since the early years of its discovery. In fact, the apparent molecular weight of the protein, as detected by SDS-PAGE, changes upon treatment with the enzyme PNGase F, which removes oligosaccharides from glycoproteins [3,13,14]. The roles of glycosylation in the secretion of serum carnosinase were described previously in Section 1.3. Elevated excretion can enhance carnosinase activity, but evidence that glycosylation also increases enzyme activity was collected both in cell models and in vivo [31]. Carnosinase activity was also higher in tissue homogenates of animal models of diabetes, which is a disease where a higher degree of enzyme glycosylation is expected [53].

Interestingly, carnosine hydrolysis, as induced by human serum or by the recombinant enzyme, has superimposable kinetics [39]. This implies that serum carnosinase can be considered as a major contributor to carnosine degradation, and any other serum enzyme able to operate carnosine hydrolysis cannot kinetically compete with serum carnosinase. For this reason, the activity of the human enzyme can be conveniently studied by using human serum, without enzyme purification. Further support for this claim comes from the evidence that different analytical methods gave consistent results concerning the enzymatic activity of whole human serum, which remains in a range between 0.9 and 1.3 nmol/h of carnosine hydrolyzed by one microliter of serum [13,39,46,54]. This is surprising since many factors have been reported to affect the serum hydrolysis rate by influencing enzyme excretion and activity [4,24,26,27,30,32,33,37,49,55,56,57].

## 2. Compounds Able to Alter the Activity of Human Serum Carnosinase

The dipeptidase role of human serum carnosinase was described in Section 1, along with some structural features that have been found to influence its enzymatic activity (e.g., enzyme glycosylation and dimerization). However, the enzyme activity has been reported to be influenced either positively or negatively upon interaction with several compounds. Such molecules can function as competitive inhibitors or allosteric modulators. Moreover, other compounds have been reported to alter the enzyme activity, with little or no investigation of the modulation mechanism. Such compounds are mostly cations or metal alkylants, so their mode of action is likely interference with zinc binding, which is essential for enzyme activity, as reported in Section 1.5.

### 2.1. Competitive Inhibitors

Since serum carnosinase catalyzes the hydrolysis of more than one dipeptide, carnosine hydrolysis can be prevented through substrate competition. The coadministration of carnosine and other substrates was proposed as one way to circumvent carnosine hydrolysis. For instance, carnosine was found at higher concentrations in human blood when ingested with anserine [58,59]. This is probably due to the saturation of serum carnosinase caused by the simultaneous presence of two substrates. Such results are also supported by a recent in silico model, predicting anserine as a potential competitor of carnosine for the catalytic pocket of serum carnosinase [7].

The experiments aimed at inducing detectable plasmatic levels of carnosine in humans can be considered a partial replication of the pharmacokinetics profile observed in animal models [47,60,61]. Such studies address the hypothesis that the improved bioavailability of carnosine can replicate the beneficial effects observed in animal models in humans (see Section 3). 

In addition to anserine, competition studies using homocarnosine and carnosine have been reported as well [13,62]. However, anserine and homocarnosine cannot be considered as true enzyme inhibitors, since they are actual enzyme substrates. Concerning the results of the competition of homocarnosine and carnosine, some concerns must be raised on the accuracy of the measured hydrolysis rates. Such data have been collected by the fluorimetric detection of histidine [39]. However, such an amino acid is produced upon the hydrolysis of both carnosine and homocarnosine [52]. Therefore, such a method seems inappropriate to detect the carnosine hydrolysis rate, since the amount of histidine produced is generated from both substrates.

Carcinine is another naturally occurring dipeptide tested for its ability to compete with carnosine [46]. Such a compound has the same structural features of carnosine, except for the absence of a C-terminus carboxylic group. Therefore, the evidence that carcinine is not able to compete with carnosine reinforces the importance of the C-terminus carboxylic group for substrate recognition (see Section 1.5).

Interestingly, some conjugates of carnosine have been reported as compounds able to inhibit carnosine degradation. One study reported a slight inhibition in serum carnosinase induced by a trehalose derivative of homocarnosine [62]. Since such a molecule is stable to serum carnosinase, the compound can be considered a proper enzyme inhibitor. A separate study identified the monoacetyl derivative of diaminopropionyl-L-histidine as a carnosine-resistant molecule able to inhibit carnosine hydrolysis [42]. However, as for the trehalose derivative of homocarnosine, such findings were not reported in the context of a study intended for the development of serum carnosinase inhibitors. In fact, no further optimization of such compounds has been reported, and the competitive nature of inhibition is presumptive and can be speculated as only owing to the structural similarities of the compounds with carnosine.

**Figure 6:**
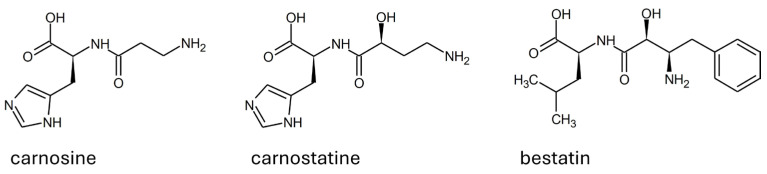
Structure and configuration of the main substrate (i.e., carnosine) and of the competitive inhibitors of human serum carnosinase.

On the contrary, carnostatine (see Figure 6) was identified as a selective and potent inhibitor of serum carnosinase through the screening of a library of thousands of protease-directed small molecules [54]. The research was intended for the development of a proper inhibitor of serum carnosinase. The nature of carnostatine as a competitive inhibitor was demonstrated in mice, along with its pharmacokinetics profile. A transgenic mouse expressing the human CNDP1 gene was used to demonstrate that a sustained inhibition of serum hydrolytic activity can be achieved by using carnostatine, without negative effects on the central nervous system or other areas [54]. In a separate study, the mechanism leading to the competitive inhibition of carnosinase 1 was explained in silico as a perturbation of the hydrolytic mechanism exerted by the hydroxyl group near the peptide bond [12].

In addition to carnostatine, bestatin is the only other inhibitor of serum carnosinase reported. Such a molecule is a protease inhibitor that is well known and tested on multiple targets [63]. As reported in Figure 6, bestatin shares the structural features described as important to determine carnostatine inhibition (i.e., a hydroxyl group in alpha to the peptide bond). During the early stages of the characterization of carnosinase-1 and its distinction from carnosinase-2, bestatin was reported as a molecule able to inhibit both enzymes in rodents [21].

Evidence of the inhibition of carnosinase-2 was also collected for the human orthologue [64]. Moreover, the crystal structure of the enzyme complex with bestatine was collected both for human and mouse carnosinase 2. Since mouse carnosinase 2 binds both zinc and manganese, the crystal structure of mouse carnosinase 2 complexed with zinc and bestatin is available (see Figure 5). Such a crystal structure reveals the key interaction of zinc with the hydroxyl group in alpha to the peptide bond, as reported in silico for carnosinol [12].

If, on the one hand, the inhibition of carnosinase 2 exerted by bestatin gave consistent results, the activity of human carnosinase 1 provided, on the other hand, controversial results. Specifically, inhibition was detected by some authors only when cadmium was added, while other authors identified a partial inhibition in human carnosinase 1 without cadmium [3,14]. Recent studies confirmed that bestatin is a competitive inhibitor for human carnosinase 1 [52]. Interestingly, the inhibition curve was characterized by a Hill slope coefficient, expected for a system where the complex between the enzyme and one molecule of the inhibitor maintains enzymatic activity. Such behavior is expected for serum carnosinase since the enzyme is dimeric (see Section 1.5) and the occupation of both binding pockets is required for complete inhibition [7,10,52].

### 2.2. Allosteric Modulators

Serum carnosinase activity was found to be regulated by compounds not able to bind the catalytic pocket but acting on allosteric sites.

For instance, during the first studies performed on the enzyme, both citrate and phosphate were found to promote carnosinase activity [4]. However, no further studies were performed, and a partial in silico explanation of the allosteric effect of citrate was collected only after a few decades, without conclusive results [11]. 

The most important results on the allosteric modulation of serum carnosinase have been collected by studying the effects on the enzyme activity of low-molecular thiols (i.e., cysteine, glutathione, N-acetylcysteine). Such compounds were found to inhibit recombinant serum carnosinase [53]. Notably, while glutathione was able to inactivate the enzyme, its disulfide form that does not have any free thiol moiety (i.e., GSSG) exerted no effect on the enzyme activity. In the same study, mutants were produced by replacing enzyme cysteine residues with serine. The experiments concluded that the thiol of cysteine102 is important for the enzyme activity. A mechanistic explanation was described in silico as a conformational change occurring upon the formation of mixed disulfides between low-molecular thiols and cysteine102 (i.e., enzyme thiolation) [53].

The allosteric role of cysteine102 is sustained by another study reporting that an increase in activity can be achieved in the recombinant enzyme upon incubation with methylglyoxal or a Fenton reagent [65]. On the contrary, decreased activity was produced by incubation with linsidomine, a molecule that was detected to induce the nitrosylation of all the cysteine of serum carnosinase, including cysteine102.

Fenton reagents are a series of chemicals mimicking endogenous processes, producing hydroxyl radicals able to damage biological substrates [66]. Methylglyoxal is an endogenous byproduct of the oxidative degradation of sugars and is involved in the cytotoxicity associated with oxidative stress [67,68,69]. Such reactions are associated with human disease development, along with other reactions determining the chemical modifications of protein and DNA, known as carbonylation [70].

Similarly, protein nitrosylation is linked to the redox regulation of biologic functions, and its deregulation is associated with human disease development [71].

The same study reporting altered activity upon the incubation of a recombinant enzyme with methylglyoxal, the Fenton reagent, and linsidomine also provided consistent results in vivo in a diabetic mice model. Specifically, the carnosinase activity in kidney homogenates was found to correlate with enzyme carbonylation and a reduction in nitrosylation associated with diabetes [65]. Importantly, the experiments reported the same level of glycosylation of the enzyme in diabetic and control mice. This finding was important to determine that carbonylation and nitrosylation were the processes responsible for the changes in the enzyme activity. In fact, as reported in Section 1.5, enzyme glycosylation has an impact on enzyme activity [31]. Therefore, it was important to measure enzyme glycosylation since higher carnosinase activity is associated with increased glycosylation, as reported in other animal models of diabetes [53]. Such results are in line with the observations that aging and other conditions associated with oxidative stress correlate with an increase in human carnosinase activity, both in serum and cerebrospinal fluid [4,13,49,50].

Notably, a mutant enzyme obtained by replacing cysteine102 with serine was reported to have lower activity, which was not influenced by incubation with methylglyoxal or a Fenton reagent [65]. All these findings reinforce the hypothesis that cysteine102 is important for the allosteric regulation of the activity of serum carnosinase.

However, other studies using inhibitor cysteine proteases gave controversial results. Specifically, the incubation with the enzyme inhibitors E64 and leupeptin did not inhibit the activity of carnosinase-1 and carnosinase-2, while p-hydroxymercurybenzoate was able to inhibit only carnosinase-2 [3]. Since E64, leupeptin, and p-hydroxymercurybenzoate are known cysteine alkylants, more studies are needed to address why thiolation or nytrosylation of cysteine102 undergo enzyme inhibition, while thiol alkylants seems not to affect the enzyme activity.

### 2.3. Compounds Interfering with Zinc

In addition to competitive inhibitors and allosteric modulators, other compounds have been reported to alter the activity of serum carnosinase. For instance, the enzyme activity is inhibited by 1,10-o-phenantrolin [3]. This is not surprising since such a compound is a metal chelator, and zinc binding is essential for enzyme activity (see Section 1.5). Evidence that metal chelators can interfere with the activity of the enzyme was reported by using EDTA. Some authors found a mild inactivation of the enzyme [4], whereas other authors reported a higher degree of inactivation [19]. Such inconsistencies may be due to the different concentrations of EDTA tested.

The activity of serum carnosinase also seems to be affected by metals. For instance, Cd(II) activates the enzyme at concentrations above 100 µM, while the activity is inhibited when the concentration of the ion is between 0.1 and 3 µM. The inhibition of activity by cadmium was associated with a reduced affinity for the substrates (i.e., carnosine and homocarnosine) and followed a dose-dependent inhibition curve when cadmium was used together with bestatin [3]. Another study reported that manganese doubled the enzyme activity, and cadmium was twice as effective as manganese [4]. On the contrary, incubation with cobalt produced 80% enzyme inhibition, while calcium and zinc had little or no effect on enzyme activity. Manganese was found to increase the enzyme stability, while cadmium and calcium reduced it. Therefore, the modulation of the activity was not fully correlated with enzyme stability. Other metals, such as Fe(II), Al(III), Co(II), and Ni(II), had no effect on enzyme activity [3]. Overall, the effect of different ions on enzyme activity can depend on the displacement or the replacement of zinc, but no specific mechanistic insights have been reported.

## 3. Human Serum Carnosinase in Human Disease

### 3.1. Human Serum Carnosinase and Central Nervous System Diseases

The roles of serum carnosinase in human disease were extensively reviewed a decade ago [72]. The first implication of such an enzyme in human health was discovered for a syndrome called carnosinemia. This name was used since a lack of carnosinase activity results in detectable levels of carnosine in the blood, unlike for healthy adults, where the activity of serum carnosinase maintains the low concentration of such a peptide [73,74]. Following studies evidenced that carnosinemia is a genetic disease. Interestingly, healthy children also have a lower serum carnosinase activity without mental deficiency or developmental delays. However, unlike for subjects affected by carnosinemia, the carnosinase activity in healthy children increases over years [75,76,77]. The implication of carnosinase activity in carnosinemia seems linked to the role of the enzyme in the central nervous system. This is supported by evidence of a correlation between reduced serum carnosinase activity and some forms of dementia, mental deficiency, and developmental delays, but also Parkinson’s disease, multiple sclerosis, and disorders associated with cerebrovascular events [73,76,78,79,80,81].

However, if, on one hand, the absence of carnosinase activity is associated with neurological disorders, on the other hand, increased enzyme activity is detrimental to the central nervous system. Specifically, the enzyme activity increases with age [50], but a faster increase in carnosinase activity is correlated with a depletion of brain carnosine and increased cognitive decline [82,83].

However, it is hard to develop animal models reproducing the effects of altered serum carnosinase activity in the human brain because such an enzyme is not found in the serum or brain of mammals other than high primates [3,14]. Moreover, in the rodent brain, the hydrolysis of carnosine is operated by the orthologue of cytosolic non-specific dipeptidase (i.e., carnosinase-2, see Section 1.2). Such an enzyme is the sole carnosinase detectable in the brain of rodents and can operate at physiological pH, unlike the human orthologue [3,18,21]. Transgenic animals expressing human serum carnosinase have been developed; however, such models have mainly been used to study the effect of carnosine metabolism in kidney diseases and not in the central nervous system [84].

### 3.2. Human Serum Carnosinase in Other Diseases

If, on one hand, some negative effects on the central nervous system have been associated with reduced or absent carnosinase activity, on the other hand, an opposite trend is reported for peripheral organs.

Some authors reported low serum carnosinase activity for athletes [38], with an apparent advantage in athletic abilities correlating with CNDP1 gene polymorphism, conducive to low enzyme activity [36,37]. This can be explained by the ergogenic effect of carnosine in the muscle, which is expected to be enhanced in subjects with reduced carnosinase activity [38,58,59,85,86]. However, physical training does not change the concentration of serum carnosinase [87], and the CNDP1 polymorphism alone is not the main factor determining the muscle concentration of carnosine, which depends mainly on age, gender, and diet [57]. Interestingly, the muscle concentration of carnosine can be enhanced upon supplementation with a parallel reduction in albumin glycation [88]. This finding is interesting since albumin glycation is a potential marker of diabetes [89]. For this reason, carnosine is used as a sport supplement, but it is also suggested as a potential therapeutic agent for the management of diabetes and cardiovascular diseases [83,90]. In this context, an enhancement in the beneficial effect of carnosine is, therefore, expected when peripheral carnosinase activity is low. This is supported by the evidence that type 2 diabetics has low levels of muscle carnosine [91].

Further hints were collected in a transgenic mouse model expressing human carnosinase-1 [92]. Specifically, carnosine supplementation and the activity of serum carnosinase both impacted the glucose metabolism and insulin secretion in such an animal model. Such evidence is consistent with the altered carnosinase activity observed in some diabetic subjects.

Interestingly, many studies associated reduced carnosinase activity with a better outcome in subjects with diabetic nephropathy [24,26,27,28,29,30,32,55,93,94,95,96,97]. Notably, kidney function and inflammation correlate with serum and urinary excretion of serum carnosinase in humans [33]. The effect observed in humans reproduces the results of animal experiments, where reduced carnosinase activity and an increased concentration of peripheral carnosine produce kidney protection, especially in diabetic nephropathy [97,98,99,100,101]. On the contrary, the overexpression of serum carnosinase was reported to aggravate diabetes and renal impairment in transgenic mice [84]. Unlike for the central nervous system, the effects observed on human and animal kidneys are similar because serum carnosinase can be found in both species [3,20,21,22,23].

In addition to chronic diseases like diabetic nephropathy or diabetes, altered carnosinase activity was also detected in humans for transient conditions such as cardiopulmonary bypass surgery [56]. One study reported a decrease in the enzyme activity during surgery, with a slow postintervention restoration. However, no further studies have been reported addressing the implications of such findings.

### 3.3. The Role of Carnosine and Homocarnosine in Diseases with Altered Carnosinase Activity

An increase in blood carnosine is likely not an etiologic factor but just a marker of disease in subjects with neurological disorders associated with low carnosinase activity. In fact, carnosine is not toxic per se, as demonstrated in several human studies where a transient increase in the carnosine concentration in blood was promoted without negative effects [58,59,102]. Conversely, carnosine’s beneficial properties have been extensively reviewed, and many authors advocate the use of such a molecule as a food supplement in sport or as a potential therapeutic agent in human disease [17,82,83,90,103,104,105,106,107]. 

Carnosine has been studied for decades in animal models, with no adverse effects and promising results in many diseases associated with an increase in oxidative stress. A few examples include atherosclerosis [61,108], diabetes [25,60], metabolic syndrome [47,109], and neurological disorders [110]. This can explain why reduced carnosinase activity in peripheral areas is associated with beneficial effects in humans, as reported in Section 3.2.

On the contrary, the adverse effects detected for syndromes such as carnosinemia are probably due to the metabolic role of the enzyme in the central nervous system. As reported in many studies, an accumulation of or reduction in homocarnosine is often associated with an altered content of gamma aminobutyric acid (GABA) and neurological effects [111,112,113,114,115,116,117]. The GABA content depends on human serum carnosinase since reduced or absent enzyme activity undergoes an accumulation of homocarnosine in the brain and cerebrospinal fluid [118]. Homocarnosine is synthesized from GABA and L-histidine and hydrolyzed by serum carnosinase (see Section 1.4). Therefore, a reduction in carnosinase activity can affect GABA recycling.

However, homocarnosine has a protective effect that resembles carnosine activity against oxidative cell damage [119,120,121,122,123,124]. Therefore, an excess of carnosinase activity can deplete the endogenous pool of homocarnosine and carnosine in the brain, which is important for the buffering capacity against oxidative stress. This can explain the association between an increase in enzyme activity, a decrease in endogenous histidine dipeptides, and increased cognitive decline [82,83]. However, the exact role of serum carnosinase as a regulator of homocarnosine and GABA levels in the central nervous system is not supported by conclusive studies.

## 4. Human Serum Carnosinase as a Druggable Target

Since carnosine has several potential applications as a food supplement or drug, some studies have focused on the development of bioavailable derivatives designed to be resistant to carnosinase while retaining activity similar to carnosine. In fact, total or partial resistance to carnosinase displays better bioavailability, as demonstrated in vivo for the natural peptides anserine and balenine. Such peptides are substrates of serum carnosinase, but unlike carnosine, they are detectable in human plasma upon supplementation [41,58,125]. However, carnosine showed better activity both in vitro and in vivo [126,127,128]. Therefore, a potential strategy to enhance carnosine bioavailability is to transport the molecule by means of drug delivery systems able to prevent the hydrolysis operated by serum carnosinase [129]. The two main strategies reported in the literature are to include carnosine in metallic nanoparticles [130,131,132] or vesicular systems [133,134,135,136].

**Figure 7:**
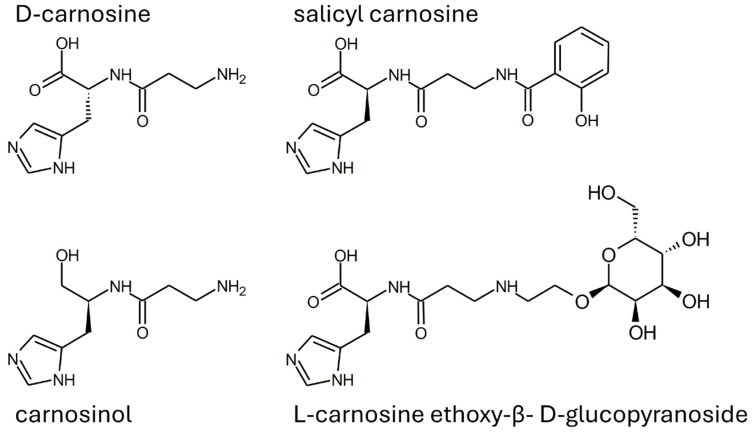
Structure of some carnosinase-resistant compounds with in vitro properties mimicking carnosine.

Alternatively, some drug design approaches have been used to obtain molecules resistant to serum carnosinase with retained or even improved carnosine-like activity. As reported in Figure 7, compounds such as carnosinol and D-carnosine were developed with small structural modifications to ensure the retention of carnosine properties as much as possible.

**Figure 8:**
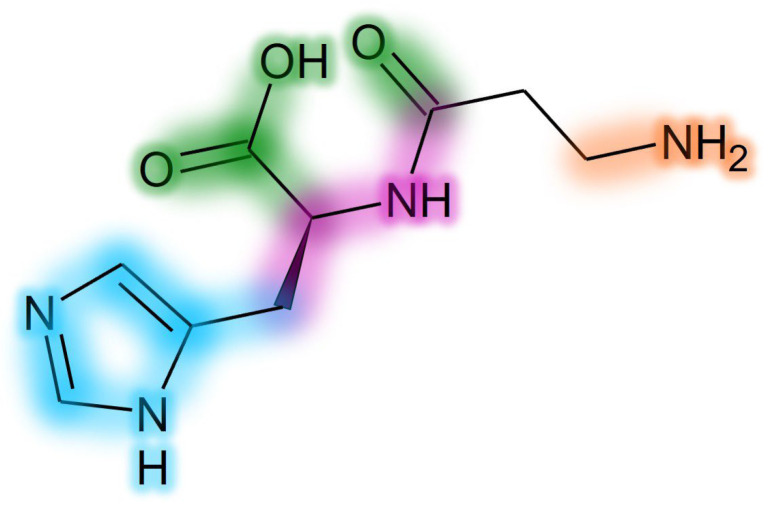
Structure–activity relationship chart for development of carnosine derivatives. Groups impacting only on carnosinase binding have a green glow. Groups impacting only on absorption (PepT1 recognition) have a purple glow. Groups impacting on both activity and carnosinase binding have a blue glow. Groups impacting on activity, absorption and carnosinase binding have an orange glow.

D-carnosine was designed to have an inversion of the configuration of the only chiral center of carnosine while retaining all other structural features. Such a molecule was as active as carnosine in vitro [47]. However, the compound required the design of prodrugs to be tested in animal models [43,108]. The reason is that the inversion of the configuration was, on one hand, good at avoiding peptide hydrolysis operated by serum carnosinase, but, on the other hand, such a structural modification was detrimental for the recognition operated by the PepT1 transporter, which is essential for carnosine absorption [17,137]. Another molecule (i.e., carnosinol) was obtained through a small modification of carnosine, namely the replacement of the carboxyl group with a primary alcohol. Unlike D-carnosine, carnosinol was readily absorbed and showed activity that was even increased, as compared to carnosine [44,45]. These findings demonstrate that the rational design of compounds resistant to serum carnosinase hydrolysis is possible. The design of carnosinol was possible since experimental data allowed for the refinement of an in silico model able to define the structure–activity relationships of carnosine derivatives [17,137]. Figure 8 shows a color-coded chart representing such a model. 

Notably, the modification of the carboxylic group of carnosine was expected to undergo a molecule stable to carnosinase, without altering the activity, as experimentally verified for carnosinol.

Other examples of compounds resistant to carnosinase have been obtained through the conjugation of carnosine with salicylic acid or sugars (see Figure 5) [138,139,140]. Such compounds have been designed since the modification of the N-terminus amine was described as a modification inducing resistance to carnosinase (see Section 1.5), in agreement with the model described in Figure 8. As recently reviewed, some of these conjugates have been designed as prodrugs able to enhance carnosine bioavailability, so they can be considered carnosine delivery systems [129].

Another appealing strategy for drug design does not involve the structural modification of carnosine but the development of inhibitors targeting serum carnosinase. This approach is an alternative to the design of compounds retaining carnosine activity but resistant to carnosinase hydrolysis. However, only one study exploiting this strategy has been reported so far [54]. In detail, the compound SAN9812, also known as carnostatine, was identified through an approach based on small-molecule library screening. The compound was tested in mice expressing the human CNDP1 gene, an animal model where carnosine cannot be detected in serum upon supplementation, like for humans. The coadministration of carnostatine and carnosine resulted in the sustained bioavailability of carnosine. As described in Section 2.1, carnostatine acts as a competitive inhibitor and was tested for its selectivity towards a panel of different enzymes and receptors, with encouraging results [54]. However, no test to detect whether carnostatine can also inhibit carnosinase-2 was performed so it is not known whether such a compound is selective towards carnosinase-1.

In addition to carnostatine, recent findings demonstrate that bestatin can also inhibit serum carnosinase, despite previous studies giving inconsistent results [52]. As for carnostatine, the inhibition is competitive (see Section 2.1). Interestingly, some data are available concerning the use of bestatin and carnosine in animal models. Specifically, bestatin was reported to have some detrimental effects in a rodent model of stroke [126]. Such effects were mitigated by the coadministration of carnosine, which also had a protective role when administered alone. Unlike carnosine, N-acetylcarnosine and anserine were ineffective. This is in line with the higher protective activity of carnosine when compared with other dipeptides with similar structures [17]. Also, these findings are consistent with the detrimental effect of the inhibition of carnosinase activity in the brain, as observed for some human genetic diseases (see Section 3.1).

Notably, bestatin inhibits carnosinase-2, which is the main enzyme in the rodent brain (see Section 1.2 and Section 2.1). Other evidence of bestatin activity was collected in a rodent model of ferroptosis. The inhibition of the cysteine transporter caused an overexpression of carnosinase-2, with implications for its glutathione metabolism and protective role. Bestatin demonstrated its ability to inhibit such an enzyme, counteracting such effects [141].

Despite such promising results, the design of a serum carnosinase inhibitor looks like a mostly unexplored field that needs more studies to further evaluate the potential applications of carnostatine and bestatin or to provide foundations for the design and development of other molecules with better properties. Overall, the data reported for carnostatine and bestatin suggest that the tissue distribution and selectivity are two key features for the successful design and development of carnosinase inhibitors. In fact, if, on one hand, peripheral inhibition looks appealing, an inhibition in carnosinase activity in the central nervous system seems dangerous. Moreover, the selectivity towards carnosinase-1 without targeting carnosinase-2 is paramount due to the different roles of the two enzymes. Unfortunately, for carnosinase inhibitors, there are no general structure–activity relationships describing which molecular features are conducive to selectivity or to the specific tissue distribution. The only structural feature that seems to be common for the inhibitors is the presence of a hydroxyl group in alpha to the peptide bond. As reported in silico for carnosinol, this can stabilize the interaction with zinc and favor the occupation of the binding pocket [12]. This model is also supported by the crystal structure of the mouse carnosinase 2 complexed with zinc and bestatin (see Figure 5). However, it is not known whether carnosinol can inhibit carnosinase 2, while bestatine inhibits both carnosinase-1 and -2. Therefore other structural features might be fundamental to develop selective inhibitors. For this reason, more experimental research is needed to gather additional information for the development of a structure–activity relationship model for carnosinase inhibitors, as already carried out for carnosine derivatives stable to hydrolysis.
